# Time evolution of Symmetry-forbidden Raman lines activated by photorefractivity

**DOI:** 10.1038/s41598-019-49801-x

**Published:** 2019-09-16

**Authors:** Ninel Kokanyan, Marco Bazzan, Laura Vittadello, David Chapron, Edvard Kokanyan, Marc D. Fontana

**Affiliations:** 1 CentraleSupélec, Université Paris-Saclay, Laboratoire Matériaux Optiques, Photonique et Systèmes, 2 rue E. Belin, 57070 Metz, France; 2 Université de Lorraine, Laboratoire Matériaux Optiques, Photonique et Systèmes, 2 rue E. Belin, 57070 Metz, France; 3 Universita di Padova, Physics and Astronomy Department, Via Marzolo 8, 35131 Padova, Italy; 4 Armenian State Pedagogical University after Kh. Abovyan, 17 Tigran Mets Ave., Yerevan, Armenia; 5 Institute for Physical Research, National Academy of Science of Armenia, Ashtarak-2, 0203 Armenia

**Keywords:** Optics and photonics, Physics

## Abstract

Transmission Raman spectroscopy experiments were performed on iron doped congruent lithium niobate within two –in principle equivalent- configurations, namely *Y*(*ZX*)*Y* and *Y*(*XZ*)*Y*. While the former respects the Raman selection rules, the other configuration gives a time dependent spectrum that, after a transient time of several minutes, finally results in a mixture of expected and forbidden modes. This breaking of Raman selection rules is caused by the spontaneous conversion of a part of the ordinarily polarized pump beam into an extraordinarily polarized beam by photorefractive anisotropic self-scattering. A numerical modelling of the phenomenon is developed and fairly reproduces the time dependence of conversion energy.

## Introduction

Lithium niobate (*LN*) has various advantageous properties such as large nonlinear optical coefficients, high transparency in the visible and near infrared range, technology for the manufacturing of waveguides^1,2^ and domain structures^3,4^. It is widely used in various applications^5,6^ such as light modulation^7^, frequency conversion^8^, *SAW* sensors^9^, photonic devices^10^. Lithium niobate, especially when doped with iron (*Fe*) shows another very remarkable property which is photorefractivity (*PR*)^11–13^. The effect is observed in the illuminated region of the crystal, and relies on the spatial transfer of photo-excited carriers, which are then re-captured in dark regions by deep traps. The result of this light-induced charge redistribution is the creation of an internal space charge field that locally modulates the refractive index of the crystal via the electro-optic effect.

Raman spectroscopy^14^ is a nondestructive method for characterizing the molecular vibrational and chemical bond structure of materials. This technique was widely used to characterize *LN* crystals with different compositions^15–17^. The assignment of all frequencies of *TO* and *LO* components of *E* and *A*_1_ phonons is now clearly established^18^. This attribution was corroborated by several ab initio lattice dynamical methods^19–21^. Just a few papers are concerned by the relationship between Raman spectroscopy and non linear optical or photorefractive properties^22–24^. Thus it is known since several decades that *PR* induces a laser defocusing which could be detected in Raman spectroscopy as a decrease in the intensity of the spectrum^22^. Later Giulotto *et al*.^23^ from one side, and Mouras *et al*.^24^ from the other side have pointed out the relaxation of Raman selection rules within 90° scattering geometry, and attributed this change to *PR*.

More recently we performed Raman measurements on *Fe*-doped *LN*^25^ in the common backscattering geometry i.e. the incident and the scattered light beams are nearly contra-propagating, so that the prime surface of the sample is analyzed. We reported a noticeable shift of Raman line with time, which was interpreted as caused by the strain associated with the space charge field *E*_*sc*_. Here we extend our investigation to deeply study the interaction between the space charge field and laser field along the propagation direction. We therefore use the transmission geometry and look at the Raman signal collected at the rear side of the sample. In addition we specially pay attention to the time-dependent change of Raman spectra. In particular, we consider two experimental conditions i.e. *Y*(*XZ*)*Y* and *Y*(*ZX*)*Y* configurations within usual Porto notations, where *XZ* and *ZX* are in principle equivalent from the point of view of the Raman selection rules. In fact, from symmetry arguments, the expected Raman spectrum for both those configurations should contain only the signature of *E*[*TO*] modes^18^. Instead, we found that one of the two equivalent configurations produces, after a transient time of the order of few minutes, a completely different Raman spectrum clearly displaying both $E[TO]$ and ${A}_{1}[TO]$ modes. This can be interpreted as the partly conversion of ordinary polarization into the extraordinary polarization. We attempt a quantitative modelling of this phenomenon and in particular of its temporal dependence.

## Experimental Details and Results

The Fe:LN crystal sample studied here was grown by the Czochralski method from a congruent melt with concentration of $0.03\,wt \%$ *Fe*. Iron ions were added to the melt in the form of $F{e}_{2}{O}_{3}$ oxide. The sample was prepared in an optical-grade parallelepiped with a thickness of $4.7mm$ along the beam propagation direction and treated at $1050\,^\circ {\rm{C}}$ for 5 hours in oxidizing atmosphere to improve the transparency. The optical absorption of the sample was measured with a *JascoV*670 spectrophotometer to be $\alpha =0.75\,c{m}^{-1}$. Transmission Raman scattering measurements were performed with a laser source at $532\,nm$ and a fixed power of $18\,mW$. The *TEM*_00_ laser mode was collimated with the help of a set of lenses to create a waist radius and transmitted through the sample.

The incident beam is propagating along the crystal *Y* axis, and can be polarized along the *Z* or *X* axis by using a *λ*/2 waveplate. An iris diaphragm is placed behind the sample to define the total angular acceptance of the light scattered within the sample. The transmitted beam is passed through a polarizer crossed with respect to the incident polarization, collected by an appropriate optics and measured by a spectrometer HORIBA Jobin Yvon iHR320. An edge filter cutting at $200\,c{m}^{-1}$ was utilized to remove the strong excitation line. The spectral resolution of the spectrometer is around $2\,c{m}^{-1}$. The configurations in trans Raman scattering *Y*(*ZX*)*Y* and *Y*(*XZ*)*Y* were successively recorded and analyzed. The far-field image of the transmitted beam was recorded as well within both configurations. For this, the image of the emerging light was projected on a screen at a distance of about 35 *cm* behind the rear face of the sample. Both the Raman and far-field pattern measurements were performed as function of time under a constant pump power. For comparison of Raman data, the same sample was also measured in the usual backscattering $Y(ZX)\bar{Y}$ and $Y(XZ)\bar{Y}$ configurations using a HORIBA Jobin Yvon Aramis spectrometer with a spectral resolution of about $1\,c{m}^{-1}$. Only $E[TO]$ modes are expected in all these four configurations according to Raman selection rules^18^ and since the Raman tensor is symmetric, all configurations are in principle fully equivalent.

**Figure 1:**
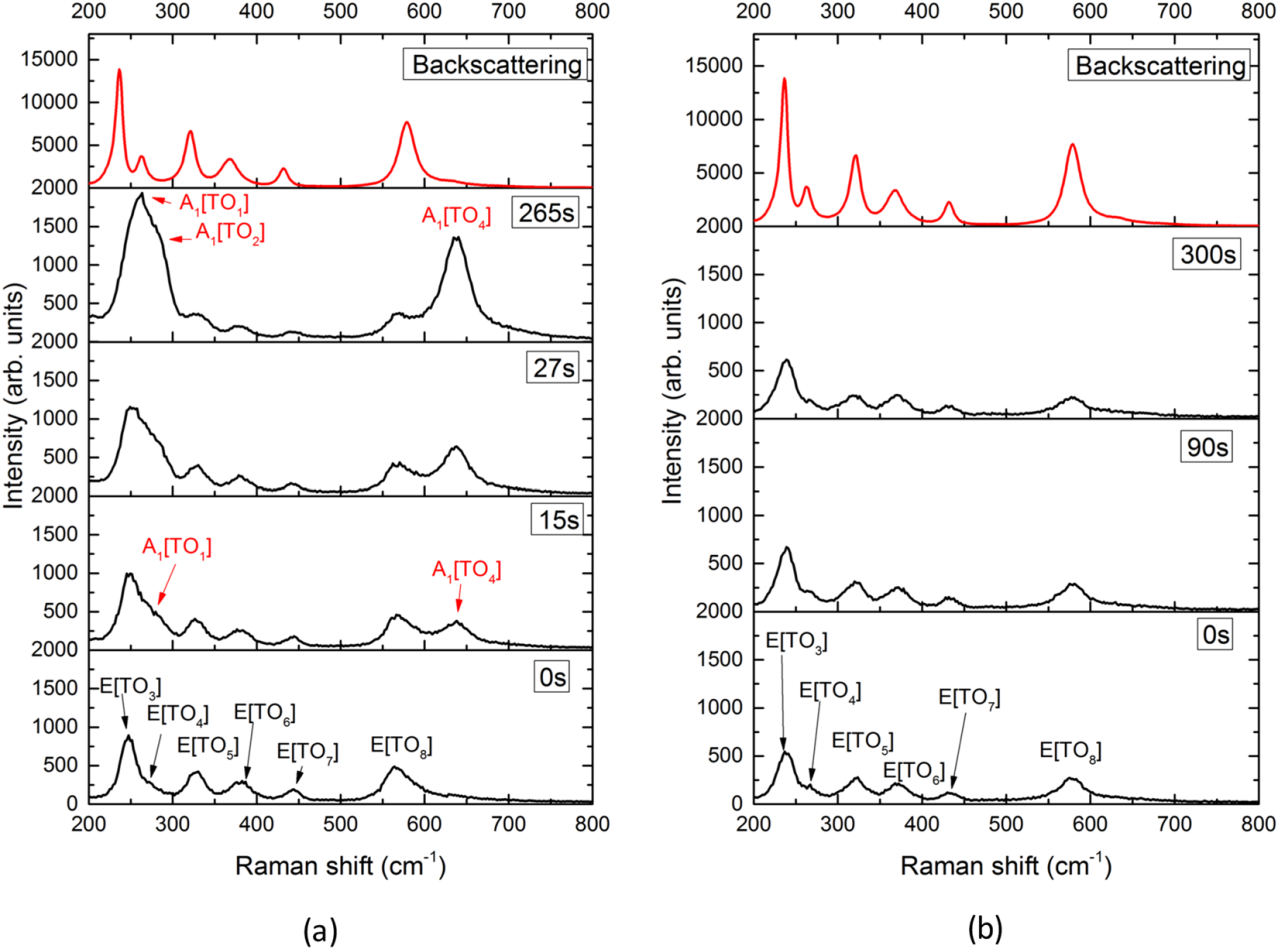
Transmission- Raman spectra as a function of time in $Y(XZ)Y$ (**a**) and $Y(ZX)Y$ (**b**) configurations for the $Fe:LN$ sample. At the top: backscattering Raman spectra independent of time are reported for comparison. We can note that the lines are any time, broader in the transmission spectra than in the back-scattering. This is due to the geometry and to the lower resolution of the spectrometer.

The transmitted Raman spectra are reported in Fig. 1 after different time intervals, and compared with corresponding back-scattered spectra, used as references. Whereas only the lines corresponding to $E[TO]$ phonons are detected at any time as expected in the $Y(ZX)Y$ configuration, the spectra in the $Y(XZ)Y$ configuration show a large evolution with time. Just at the beginning of illumination, the spectrum in the transmission geometry is very similar to the corresponding spectrum in back-scattering, but rapidly new lines appear and increase in intensity with time. After one minute of illumination, the spectrum completely differs from expected $E[TO]$. The unexpected lines cannot be attributed to the direct effect of the incorporation of iron into the lattice. First, any compositionally-induced modification of the Raman profile is expected to be static and cannot possibly explain a time-evolution on the scale of the minutes such as the one here reported. Moreover, *Fe* doping in the concentration used in this work is expected only to induce a slight change in the position and width of the Raman line^26^. These new lines in fact arise from another mode symmetry and are clearly assigned as due to ${A}_{1}[TO]$ phonons^18^. It is to be mentioned that ${A}_{1}[TO]$ modes are polarized along the extraordinary *Z* axis and normal to $E[TO]$ modes.

Generally, the detection of unexpected lines by symmetry and selection rules can arise from a small disorientation of the crystal axes with respect of lab references axes, and/or non-perfect quality of polarizer or analyzer leading to a leakage of the polarization. These reasons are fully excluded here owing to the time dependence and magnitude of the observed feature. Furthermore, the comparison between both configurations within transmission geometry discards any source of error coming from optical set up. The contrast with the back-scattering data in which only expected $E[TO]$ lines are detected is remarkable since both geometries are in principle equivalent.

**Figure 2:**
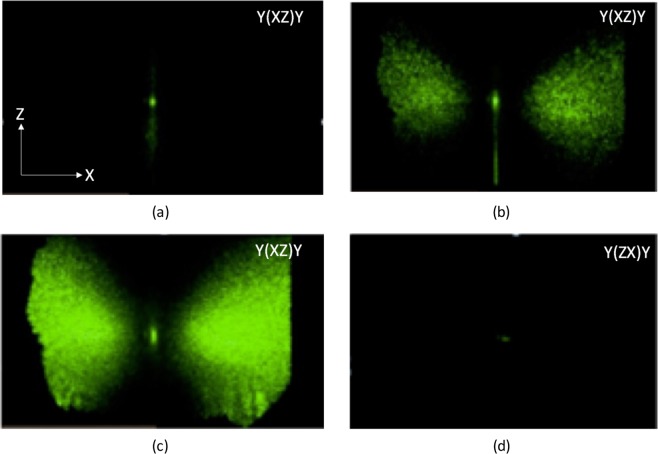
Far- field pattern in forward scattering for $Y(XZ)Y$ configuration at different exposure times: (**a**) 20 seconds, (**b**) 80 seconds, (**c**) 220 seconds. (**d**) $Y(ZX)Y$ configuration. The two arrows indicate the crystal axis directions.

The far-field pattern of the transmitted beam recorded with the pump beam polarized along *X* and the analyzer along *Z*, i.e. in the $Y(XZ)Y$ geometry at different times are presented in Fig. 2. One can notice the beam pattern evolution, showing that after some time a new light field extraordinarily polarized is produced along two lobes symmetrically placed with respect to the pump spot. This explains the activation of ${A}_{1}[TO]$ Raman lines, the intensity of which increases with time: this part of the Raman spectrum is produced by the newly generated extraordinary beams which corresponds to a $Y(ZZ)Y$ scattering geometry. The lobes are most pronounced in the *XY* plane, with a maximum at an angle of about 5 degrees with respect to the pump beam. By changing the iris diaphragm aperture placed behind the crystal it is possible to verify that the contribution of the ${A}_{1}[TO]$ Raman lines in the detected spectrum comes essentially from the scattered intensity emitted in the direction of the lobes maxima.

In contrast, the beam pattern for the $Y(ZX)Y$ configuration remains unchanged (Fig. 2). In this last case, the transmitted beam between crossed polarizers is co- linear with the incident beam (along x) and the polarization is not changed. Therefore, the activation of forbidden lines occurs solely in the $Y(XZ)Y$ geometry, and its time dependence is connected to the build-up of the extraordinary scattered beam, as the ordinarily polarized beam propagates through the PR sample.

**Figure 3:**
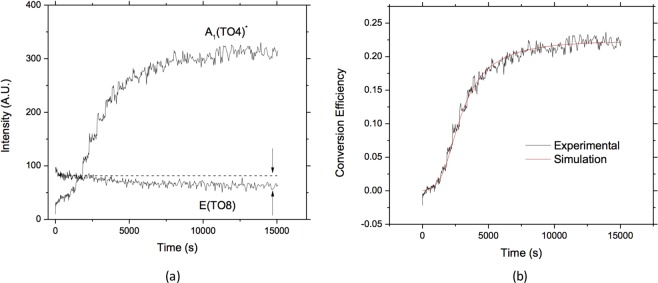
(**a**) Peak intensities of activated (forbidden) ${A}_{1}{[T{O}_{4}]}^{\ast }$ and expected $E[T{O}_{8}]$ Raman peaks in the $Y(XZ)Y$ configuration. The dashed line is a guide to the eye to show the decrease of the $E[T{O}_{8}]$ peak. (**b**) Comparison between experimental conversion efficiency (black) and the numerical simulations (red).

The origin of the activation of ${A}_{1}[TO]$ (denoted hereafter ${A}_{1}{[T{O}_{4}]}^{\ast }$) modes in the $E[TO]$ spectrum is straightforwardly related to the PR properties of $LN:Fe$ crystals. The intensity of the forbidden Raman lines reflects directly the time evolution of the conversion from the o- polarization to the e- polarization after crossing the sample. The time evolution of the two superimposed spectra is reported in Fig. 3(a) by measuring at different time intervals the height of the $E[T{O}_{8}]$ and ${A}_{1}{[T{O}_{4}]}^{\ast }$ peaks as they can be discerned more easily than others. The former, belonging to the expected $E[TO]$ spectrum, shows a decrease of about 20% from its initial value. The latter, belonging to the ${A}_{1}{[T{O}_{4}]}^{\ast }$ spectrum forbidden by Raman selection rules, shows a clear increase. Note that the intensity of the ordinary and extraordinary components have a different amplitude: this is due to a number of factors, including the different magnitude of the Raman tensor elements, the different optical path followed by the two beam and possible different efficiencies in the collection optics.

To evaluate quantitatively the phenomenon it is therefore convenient to recast it in terms of the conversion efficiency, where *P*_2_ is the power of the extraordinary beam after the sample and ${P}_{1}+{P}_{2}$ is the total beam power. If absorption can be neglected, as in our case, this latter term is equal to the power of the pump beam before the sample. Assuming that the pump beam is coupled to one single extraordinary wave propagating along the maximum intensity direction (i.e. at 5° from the ordinary beam), by energy conservation we can renormalize the signal of the forbidden Raman line so that the fraction of power gained by the extraordinary beam is equal to the one lost by the pump beam. In this way we get rid of most of the systematic errors in intensity measurements due to unknown conversion factors between the Raman signals and the measured beam powers, and to the fact that a fraction of the extraordinary signal is lost because of the limited angular acceptance of the detection system. It should be noted that measuring the ratio of the Raman lines represents a very convenient experimental approach to access the ordinary-to extraordinary conversion. This is more robust against stray light and misalignments than a basic polarization measurement, provided that the angular direction of the scattered light collected by the spectrometer is somehow defined by appropriate collection optics.

## Simulation

To analyze the dependence of the forbidden lines intensity upon time we realized a simulation code based on previous works by Wilson^27^ and Fluck^28^. Essentially, the coupling phenomenon is enabled by a transfer of power between the ordinary (pump) and the extraordinary (generated) power mediated by the following occurrences: (i) a nonzero component of the photo-galvanic tensor which, upon the simultaneous presence of two orthogonally polarized beams with differing wavevectors, produces a spatially modulated current and in turn a space charge field (ii) a nonzero component of the electro-optic tensor, which transforms the spatially modulated field into a refractive index grating diffracting the pump beam into the generated one. Note that the refractive index term (in Voigt notation) responsible for the coupling is nondiagonal, so that the ordinary beam is diffracted into an extraordinary one. The phenomenon is therefore self-starting: any weak extraordinary seed with sufficient intensity^29^ initially present into the sample produces the modulated photogalvanic current, which in turns amplifies the extraordinary beam itself and so on, until a saturation regime is attained. The problem is to describe the spatio-temporal evolution of the probe beam and of the PR –generated beam as a function of the position and the time.

**Figure 4:**
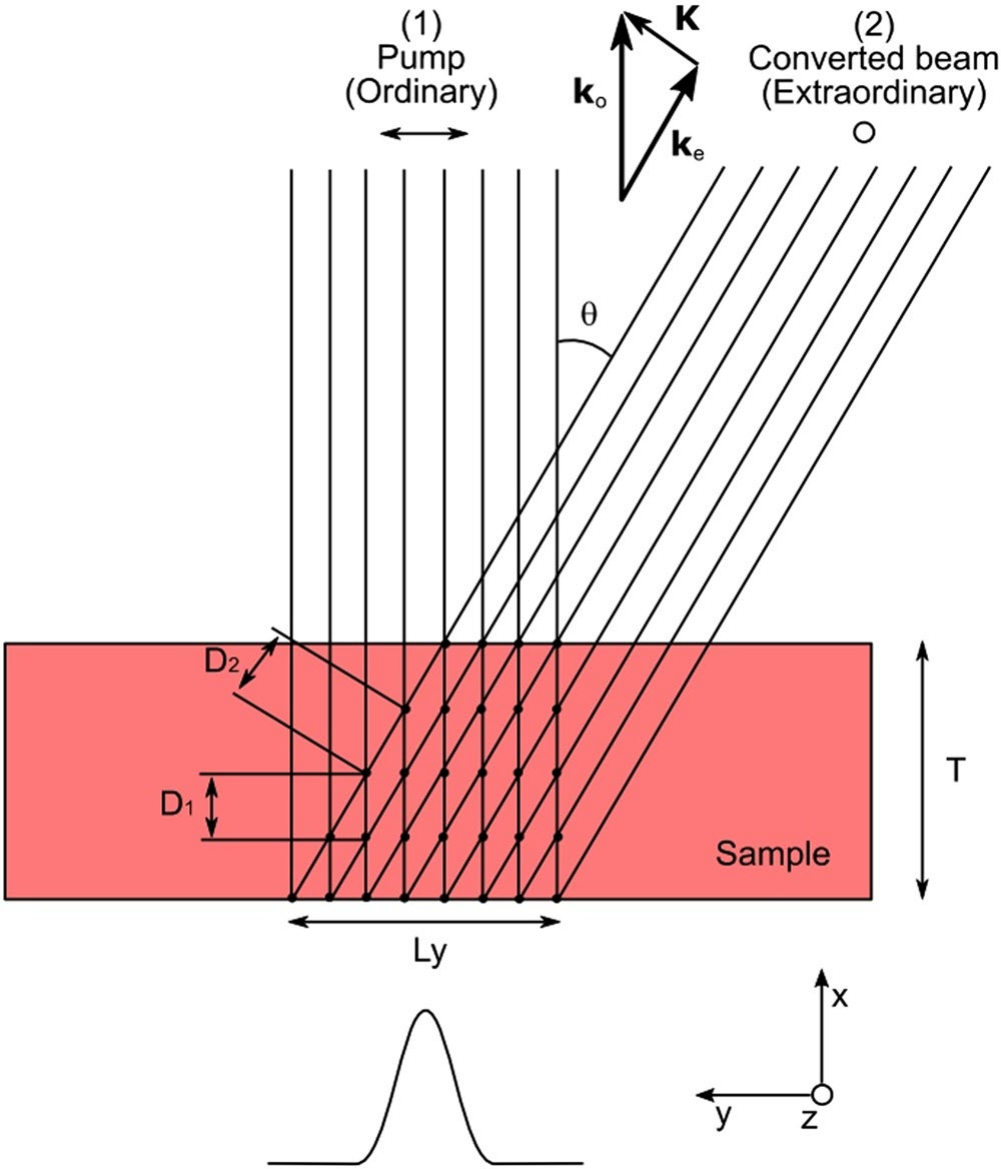
Scheme adopted for the simulation of the photorefractive anisotropic self-scattering process.

Let us consider the experimental situation sketched in Fig. 4. The Raman probe at 532 *nm* is described by a Gaussian function characterized by the waist and by the power. Since our typical sample thickness is much smaller than the Rayleigh range of our transmitted probe beam (several cm), the intensity of the pump beam can be described along a set of parallel lines. As shown in Fig. 2, the generated extraordinary beam has an intensity that depends on the direction with respect to the primary beam. In the following, for sake of simplicity, we will consider that the extraordinary beams are emitted only in one specific direction, i.e. in the plane xy at an angle theta with the primary beam corresponding to the direction along which the scattered intensity is maximized. In this way we can build the grid reported in Fig. 4 where the evolution of the two beams can be computed numerically as a function of the position on each node and for any time interval.

The power transfer between the two polarization states inside a photorefractive crystal is described by the following coupled differential equations^27^:

1 $$\begin{array}{rcl}\frac{\partial {I}_{2}}{\partial {x}_{2}} & = & {C}_{12}\frac{{I}_{1}{I}_{2}}{{I}_{1}+{I}_{2}}-\alpha {I}_{2}\\ \frac{\partial {I}_{2}}{\partial {x}_{1}} & = & -\,2{C}_{12}\frac{{I}_{1}{I}_{2}}{{I}_{1}+{I}_{2}}-\alpha {I}_{1}\end{array}$$

where ${I}_{1(2)}$ are the intensities and ${x}_{1(2)}$ the propagation directions of the two beams, *α* is the absorption coefficient and *C*_12_ is a coupling coefficient. The factor 2 visible in the second line is due to the fact that the pump power is depleted by the two symmetrically displaced extraordinary beams. The coupling coefficient *C*_12_ depends on the position and on time, according to the following equations:

2 $${C}_{12}(x,y,z,t)=\Gamma f(x,y,z,t)D(x,y,z,t)S(x,y,z,t)$$

3 $$(\frac{{\tau }_{0}}{{I}_{1}+{I}_{2}}\frac{\partial }{\partial t}+1)\,f=1$$

4 $$D=\frac{1}{1+{I}_{dark}/({I}_{1}+{I}_{2})}$$

5 $$S=\{\begin{array}{ll}1 & inside\,the\,sample\\ 0 & elsewhere\end{array}$$

In Eq. (2) the coefficient $\Gamma$ is the photorefractive gain which is material constant with the dimensions of *m*^−1^. Photorefractivity theory gives^27^:

6 $$\Gamma =2[\hat{K}\widehat{{e}_{o}}]\frac{\omega {n}_{o}{n}_{e}{r}_{51}}{{c}^{2}{\varepsilon }_{0}}{E}_{sat}$$

where $\hat{K}$ and $\widehat{{e}_{o}}$ are unitary vectors parallel to the wavevector difference $K={k}_{o}-{k}_{e}$ and to the ordinary polarization direction respectively. $\omega$ is the optical frequency of the pump beam, ${n}_{o(e)}$ are the ordinary (extraordinary) refractive indexes at the pump wavelength, *c* is the speed of light and ${\varepsilon }_{0}$ is the vacuum permittivity. *r*_51_ is the electro-optic coefficient and *E*_*sat*_ is the magnitude of the space charge field component (directed along *X*) in stationary conditions, after the transient has ended. Apart from the latter, all the other parameters are a priori known. The square bracket factor in Eq. (6) is responsible for the angular dependency of the scattered intensity. It should be noted that this factor show a quick increase for *θ* between 0 and 4 degrees, and then remains almost constant in a wide angular range (in agreement with our data in Fig. 2). In the following we set therefore $\theta =5^\circ$.

In Eq. (3) ${\tau }_{0}$ is the time constant of the PR process. Equations (3 and 4) govern the space and time evolution of the coupling constant, as they are dependent on the beam intensities. Here we make substantial use of a “local” approximation which states that, for slowly varying intensities, the photorefractive equations for uniform illumination remain valid provided that intensity-dependent quantities are replaced by their local value^28^. Thus, the term $\frac{{I}_{1}+{I}_{2}}{{\tau }_{0}}$ in Eq. (3) has the dimensions of *s*^−1^ and corresponds to the “local” Maxwell relaxation time of the system. If the beam intensities were constant, this term would remain equal everywhere and the solution to Eq. (3) would be the familiar saturating exponential function. Equation (4) describes the effect of the material background conductivity. It plays a role of fundamental importance since without it, for sufficiently long times, all the points of the sample would reach the saturation gain coefficient $\Gamma$. Instead, for local intensities ${I}_{1}+{I}_{2}$ much smaller than the *I*_*dark*_ value, the coupling coefficient remains small and proportional to ${I}_{1}+{I}_{1}/{I}_{dark}\ll 1$. Finally, Eq. (5) confines the possibility of a coupling only inside the sample. In summary, the time evolution of the extraordinary beam intensity is described by three free parameters: *E*_*sat*_, ${\tau }_{0}$ and *I*_*dark*_, all the other parameters being known or independently measurable.

We wrote a dedicated simulation software that solves Eqs (1–6) on the discrete grid reported in Fig. 4. The initial conditions are given by the input pump beam and by a uniform seed, assumed to have everywhere the arbitrary initial intensity of ${10}^{-6}\,W/{m}^{2}$. We verified that other choices of this value do not affect the simulation results, as long as it remains much weaker than the pump beam. First, the code computes *I*_1_ and *I*_2_ on all the points of the grid. Then, the time evolution f for all the nodes is computed from the discrete version of Eq. (3) by using a suitably chosen time step. The other intensity- dependent quantities are computed using Eqs (2) and (4). The code then calculates the new intensities on each node, solving iteratively the discrete version of Eq. (1):

7 $$\begin{array}{rcl}{I}_{2}(i+1,j,k) & = & {I}_{2}(i,j,k)+{C}_{12}(i,j,k)\frac{{I}_{1}(i,j,k){I}_{2}(i,j,k)}{{I}_{1}+{I}_{2}}{D}_{2}\\  &  & -\,\alpha {I}_{2}(i,j,k){D}_{2}\\ {I}_{1}(i,j+1,k) & = & {I}_{1}(i,j,k)-2{C}_{12}(i,j,k)\frac{{I}_{1}(i,j,k){I}_{2}(i,j,k)}{{I}_{1}+{I}_{2}}{D}_{1}\\  &  & -\,\alpha {I}_{1}(i,j,k){D}_{1}\end{array}$$

where ${D}_{1(2)}$ are the propagation steps in the two beams directions, as shown in Fig. 4. The procedure is then iterated for a sufficiently long time, until the system reaches the saturation. The powers of the transmitted (pump) beam 1 and of the converted beam 2, *P*_1_ and *P*_2_ respectively, are calculated by integrating the beam intensities *I*_1_ and *I*_2_ on the output face of the crystal for each time step. We can finally compute the conversion efficiency by calculating as a function of time $\eta =\frac{{P}_{2}}{{P}_{1}+{P}_{2}}$.

The simulation parameters *E*_*sat*_, ${\tau }_{0}$ and *I*_*dark*_ best reproducing the experimental curve were determined for our sample by a trial-and error procedure. It is important to stress that this monotonic saturation curve has a quite complicated behavior: initially the conversion efficiency is small and increases slowly, then it speeds up and finally reaches a stationary value. The corresponding results are shown in Fig. 2 for ${E}_{sat}=(1.65\pm 0.30)\times {10}^{3}\,V/m$, ${\tau }_{0}=(2.6\pm 0.2)\times {10}^{7}\,sW/{m}^{2}$ and ${I}_{dark}=4000\pm 1000\,W/{m}^{2}$, where it appears clearly that our simulation code is able to reproduce satisfactorily the full dynamics of the spectrum evolution. The first parameter, *E*_*sat*_ determines the amplitude of the forbidden Raman peaks at the end of the temporal evolution, while ${\tau }_{0}$ describes the duration of the transient. The parameter *I*_*dark*_ affects both the amplitude and the evolution speed of the curve, so it is partially interdependent on the other two parameters. However, the first part of the curve between 0 and 1000 s is only weakly affected by the choice of *I*_*dark*_. This fact allows finding a unique combination of parameters providing a satisfactory agreement.

The incertitude reported in the simulation parameters concerns only the random errors and is estimated taking into account the mentioned partial dependency among them, as well as the fact that the data to be fitted are obtained through the renormalization procedure described in Section 2. In particular, it is especially important to determine accurately the depletion of power from pump beam 1 as this sets the saturation value of the curve in Fig. 3b and thus the final value of *E*_*sat*_. A systematic error source is due to the fact that in our simulation for simplicity the extraordinary beam is treated as a uni-directional plane wave directed at 5° from the ordinary one. A more accurate treatment would consist in taking into account the angular dependence of the scattered beams and in calculating the evolution of the extraordinarily polarized intensity as an average over the angular range accepted by the detector. However, as discussed above, in the range between 4° to about 20°, the angular dependence of the gain coefficient (Eq. 6) is weak, while between 0 and 4 degrees this quantity goes quickly to zero, so that the signals coming from this range are contributing in a limited amount to the final curve. The overall relative error in performing our simplified treatment is thus expected to be in the range of 10–20% depending on the parameters.

## Conclusion

Transmission Raman spectra recorded in photorefractive iron-doped *LN* crystal within a priori equivalent configurations, $Y(XZ)Y$ and $Y(ZX)Y$ are completely different as function of time. In $Y(ZX)Y$ only $E[TO]$ modes are present in accordance with selection rules, while in $Y(XZ)Y$ configuration spectra show a strong dependence on time with a rise of forbidden ${A}_{1}[TO]$ Raman modes. The intensity of the forbidden activated lines reveals the time evolution of the conversion from the o- polarization to the e- polarization after crossing the sample. The intensity ratio of activated ${A}_{1}{[T{O}_{4}]}^{\ast }$ and $E[T{O}_{8}]$ reflects the conversion efficiency as a function of time. This time dependence is fairly well reproduced by simulations based upon the model earlier proposed by Wilson *et al*. These calculations provide an estimation of the parameters characterizing the photorefractive effect and were discussed in terms of sources of random and systematic errors. We stress that the photorefractive parameters *E*_*sat*_, ${\tau }_{0}$ and *I*_*dark*_ can be directly related with the help of standard photorefractivity theory (see e.g. ref. ^6^) to sample characteristics, such as [*Fe*] concentration and reduction degree. Our study combining experimental Raman measurements and simulations therefore opens a new way to investigate photorefractive photonics by using transmitted Raman spectroscopy.
